# Using Large Language Models to Detect and Understand Drug Discontinuation Events in Web-Based Forums: Development and Validation Study

**DOI:** 10.2196/54601

**Published:** 2025-01-30

**Authors:** William Trevena, Xiang Zhong, Michelle Alvarado, Alexander Semenov, Alp Oktay, Devin Devlin, Aarya Yogesh Gohil, Sai Harsha Chittimouju

**Affiliations:** 1 Department of Industrial and Systems Engineering The University of Florida GAINESVILLE, FL United States; 2 Department of Industrial and Systems Engineering The University of San Diego San Diego, CA United States; 3 Microsoft Seattle, WA United States

**Keywords:** natural language processing, large language models, ChatGPT, drug discontinuation events, zero-shot classification, artificial intelligence, AI

## Abstract

**Background:**

The implementation of large language models (LLMs), such as BART (Bidirectional and Auto-Regressive Transformers) and GPT-4, has revolutionized the extraction of insights from unstructured text. These advancements have expanded into health care, allowing analysis of social media for public health insights. However, the detection of drug discontinuation events (DDEs) remains underexplored. Identifying DDEs is crucial for understanding medication adherence and patient outcomes.

**Objective:**

The aim of this study is to provide a flexible framework for investigating various clinical research questions in data-sparse environments. We provide an example of the utility of this framework by identifying DDEs and their root causes in an open-source web-based forum, MedHelp, and by releasing the first open-source DDE datasets to aid further research in this domain.

**Methods:**

We used several LLMs, including GPT-4 Turbo, GPT-4o, DeBERTa (Decoding-Enhanced Bidirectional Encoder Representations from Transformer with Disentangled Attention), and BART, among others, to detect and determine the root causes of DDEs in user comments posted on MedHelp. Our study design included the use of zero-shot classification, which allows these models to make predictions without task-specific training. We split user comments into sentences and applied different classification strategies to assess the performance of these models in identifying DDEs and their root causes.

**Results:**

Among the selected models, GPT-4o performed the best at determining the root causes of DDEs, predicting only 12.9% of root causes incorrectly (hamming loss). Among the open-source models tested, BART demonstrated the best performance in detecting DDEs, achieving an *F_1_*-score of 0.86, a false positive rate of 2.8%, and a false negative rate of 6.5%, all without any fine-tuning. The dataset included 10.7% (107/1000) DDEs, emphasizing the models’ robustness in an imbalanced data context.

**Conclusions:**

This study demonstrated the effectiveness of open- and closed-source LLMs, such as GPT-4o and BART, for detecting DDEs and their root causes from publicly accessible data through zero-shot classification. The robust and scalable framework we propose can aid researchers in addressing data-sparse clinical research questions. The launch of open-access DDE datasets has the potential to stimulate further research and novel discoveries in this field.

## Introduction

The development of the transformer model [1] has significantly advanced natural language processing (NLP), a key area of artificial intelligence (AI). These models, commonly referred to as large language models (LLMs), often contain billions or trillions of parameters and have greatly improved our ability to extract meaningful insights from unstructured data [2], achieving state-of-the-art results in various NLP tasks since the introduction of bidirectional encoder representations from transformers [3]. LLMs have excelled in tasks, such as translation, answering of questions, summarization, sentiment analysis, language generation, and named entity recognition. They are used in diverse fields, including analyzing corporate filings for investment decisions [4], assessing online reviews [5], and performing sentiment analysis of tweets [6].

Recently, advanced LLMs such as DeBERTa (Decoding-Enhanced Bidirectional Encoder Representations from Transformers with Disentangled Attention) [7] and OpenAI’s GPT-4 [8] have demonstrated substantial performance improvements. These enhancements include improvements in model architecture, such as DeBERTa’s disentangled attention mechanism and GPT-4’s increased model size and training data, which enhance their prediction accuracy and their capacity to handle complex language understanding tasks. These advancements have opened new possibilities for health care applications of NLP, such as analyzing social media and online forums to assess trust and confidence toward vaccine use [9], adverse drug reactions [10,11], and depression [12], among others [13-16]. However, many potential applications of NLP in health care remain underexplored.

One critical issue is early drug discontinuation and nonadherence to prescribed medication, which negatively affects patient outcomes and increases health care costs [17]. The reasons behind early medication discontinuation are not well understood [18,19], and detecting drug discontinuation events (DDEs) from clinical narratives and social media data is underexplored.

Some studies have explored medication discontinuation using data from social media and NLP techniques. Golder et al [20] conducted a mixed methods study analyzing patient-reported reasons for switching or discontinuing statin therapy using social media data from WebMD. Their work focused specifically on statins and relied on manual annotation for classifying DDEs and their root causes. Similarly, van Buchem et al [21] developed and validated the AI patient-reported experience measure (AI-PREM) by analyzing patient experiences using NLP. Their study primarily used sentiment analysis to gauge patient experiences but did not focus on detecting specific events such as DDEs.

Another study by Tsai et al [22] used NLP and network analysis to examine patients withdrawing from life-sustaining treatments using electronic health records from 119 patients in Taiwan. Their approach involved generating document-term matrices and analyzing word frequencies to identify patterns. However, they did not use pretrained LLMs, and their study was limited to a small dataset.

While these studies and others [23-26] contribute valuable insights into patient experiences and reasons for medication discontinuation, they have limitations such as focusing on specific medications, relying on manual annotation, or not leveraging advanced LLMs for broader applicability. To the best of our knowledge, no prior studies have systematically analyzed the root causes of medication discontinuation using state-of-the-art LLMs such as OpenAI’s GPT-4o, especially in a zero-shot classification setting applied to large-scale, unstructured patient-generated data from web-based forums.

Historically in many NLP applications, the models needed to be fine-tuned or adjusted for each specific task. However, state-of-the-art LLMs are capable of zero-shot classification, which are especially valuable in settings where data are scarce, as they do not require modifications or fine-tuning on task-specific data. This feature is particularly beneficial in the medical field, where access to data is often limited due to patient confidentiality concerns. Motivated by challenges associated with limited data availability in health care research, the primary aim of this study is to develop a versatile framework for addressing clinical research questions in data-sparse settings by leveraging pretrained LLMs and zero-shot classification of unstructured data from web-based health forums. The specific objectives of this paper are as follows:

1. To present a methodical approach to detecting DDEs posted on web-based health forums
2. To identify the root causes of DDEs posted on web-based health forums
3. To evaluate the effectiveness of different classification strategies and models in identifying DDEs and their root causes
4. To introduce and validate the first open-source DDE detection dataset and the first open-source DDE root cause classification dataset to support ongoing and future research

In addition, our work is novel in 4 additional aspects. First, studies in the extant work mainly used electronic health records (EHRs) as the data source [17]. Due to the poor documentation of drug discontinuation events (DDEs) in EHRs [17], it is crucial to understand the causes of discontinuation from the patient’s perspective to improve medication adherence. Traditional EHR data often lack direct patient input, as they are usually filtered through health care providers. Moreover, privacy concerns surrounding patient health data often restrict accessibility to large open-source datasets for health research. Meanwhile, web-based forums such as MedHelp offer unmediated patient perspectives, provide insights not captured in EHRs, and involve a large and diverse population. Identifying the root causes of DDEs from the patient’s viewpoint on the basis of a representative sample can enhance our understanding of patient behavior and the challenges they face.

Second, to the best of our knowledge, this is the first work that compares state-of-the-art text-to-text models with the previous generation of models that receive text as an input and respond with text as an output, such as GPT-4o. Many open-source pretrained large language models (LLMs) fine-tuned for zero-shot classification are available through platforms like Hugging Face [27]. These models can predict whether a given text supports an arbitrary hypothesis without needing additional training for each new task. This means that if a human reader can infer the truth of a hypothesis from the text, the model can make a prediction to do the same [28]. Typically, these models output a probability between 0 and 1, indicating how likely it is that the hypothesis is true based on the given text. To be able to apply these approaches to the problem, we designed a novel framework that leverages these models to identify DDE and the associated root causes.

Third, we demonstrated the effectiveness of LLMs in addressing clinical research questions without extensive training data. This approach overcomes the limitations of previous methods [17] by using transfer learning to tackle data scarcity. Our study shows that LLMs such as bidirectional and auto-regressive transformers (BART) and GPT-4o, with their generalization capabilities, are versatile for new tasks. This novel methodology could revolutionize the way clinical research questions are approached, opening a new paradigm in health informatics research.

Fourth, this paper introduces 2 open-source datasets, the first of their kind: one focused on DDE detection and another on root cause classification of DDEs. By creating and publishing these datasets, we aimed to motivate and facilitate future research efforts toward investigating drug discontinuation. For example, researchers could use these datasets to investigate why patients discontinue specific medications early. By extracting and analyzing comments that describe DDEs, they can identify the root causes and guide future research to address side effects or other factors associated with drug discontinuation.

Overall, this research provides valuable insights into patient behavior and decision-making regarding medication use, highlighting the real-world challenges faced by the patients. These insights can inform health care providers, policy makers, and pharmaceutical companies, helping them to better understand and address barriers to medication adherence.

## Methods

### Overview

The effectiveness of research outcomes in NLP relies heavily on the methods and materials used. This section outlines the strategies and resources used in this study, focusing on LLMs. In particular, this study introduces an innovative approach by redefining DDE detection as a natural language inference task. This approach enables LLMs to leverage their existing training to deduce relationships between text elements, achieving the desired outcomes without the need for additional training data or model fine-tuning.

We sourced data from MedHelp [29], a publicly accessible web-based health community where users discuss health experiences and share advice. This section details the methodology used for detecting DDEs from comments posted on MedHelp and analyzes the root causes of these DDEs using LLMs.

### Data Scraping and Preprocessing

Our dataset was compiled by scraping 759,872 questions and answers from 131 of the 193 communities on MedHelp. Each question or answer has a unique URL, with information corresponding to each being recorded as a separate row in our dataset.

The dataset was then trimmed down by excluding questions and answers that lacked relevant study keywords. These keywords were derived from the International Drug Dictionary dataset [30], reducing our dataset to 183,565 questions and answers.

### Data Labeling

#### Overview

To create the first open-source DDE datasets and to assess the accuracy of LLMs at detecting DDEs and identifying their root causes, we used graduate students to label the data obtained from MedHelp. For DDE detection, 1000 comments were labeled, resulting in 893 (89.3%) non-DDE comments and 107 (10.7%) DDE comments. Of the 89 (8.9%) comments that the labelers did not unanimously agree upon, the majority vote was taken as the “ground truth.” Here, the “ground truth” refers to the correct classification for each comment, based on the consensus of the labelers.

For DDE root cause analysis, 1000 (100%) comments suspected of containing DDEs were categorized based on predefined criteria. Multiple root causes can apply to a single DDE and a single comment can express multiple DDEs, allowing multiple root causes to be assigned to one comment. When the labelers disagreed, they discussed the comments to reach a consensus.

#### Classifying Comments as DDE or Non-DDE

Labelers were asked to categorize each comment into either of the following categories:

- 0—non-DDE (the comment does not contain a DDE)
- 1—DDE (the comment contains a DDE)

The comments were classified as DDE if a specific person’s discontinuation of a recurring medication or treatment could be inferred from it. Medication changes were considered as DDEs while one-time treatments were not. Apart from this, labelers were asked to indicate their level of confidence in their labels by selecting one of the three confidence levels: (1) very confident (2890/3000, 96.33%); (2) somewhat confident (102/3000, 3.4%); and (3) not confident (8/3000, 0.27%). The percentage in parentheses alongside each confidence level indicates the rate at which the confidence level was selected by the labelers.

#### Classifying the Root Causes of DDEs

After identifying comments containing DDEs, the next step involved categorizing the root causes of these discontinuations. Labelers were provided with detailed guidelines and definitions for each category to ensure consistency and accuracy (Textbox 1).

**Figure 1:**
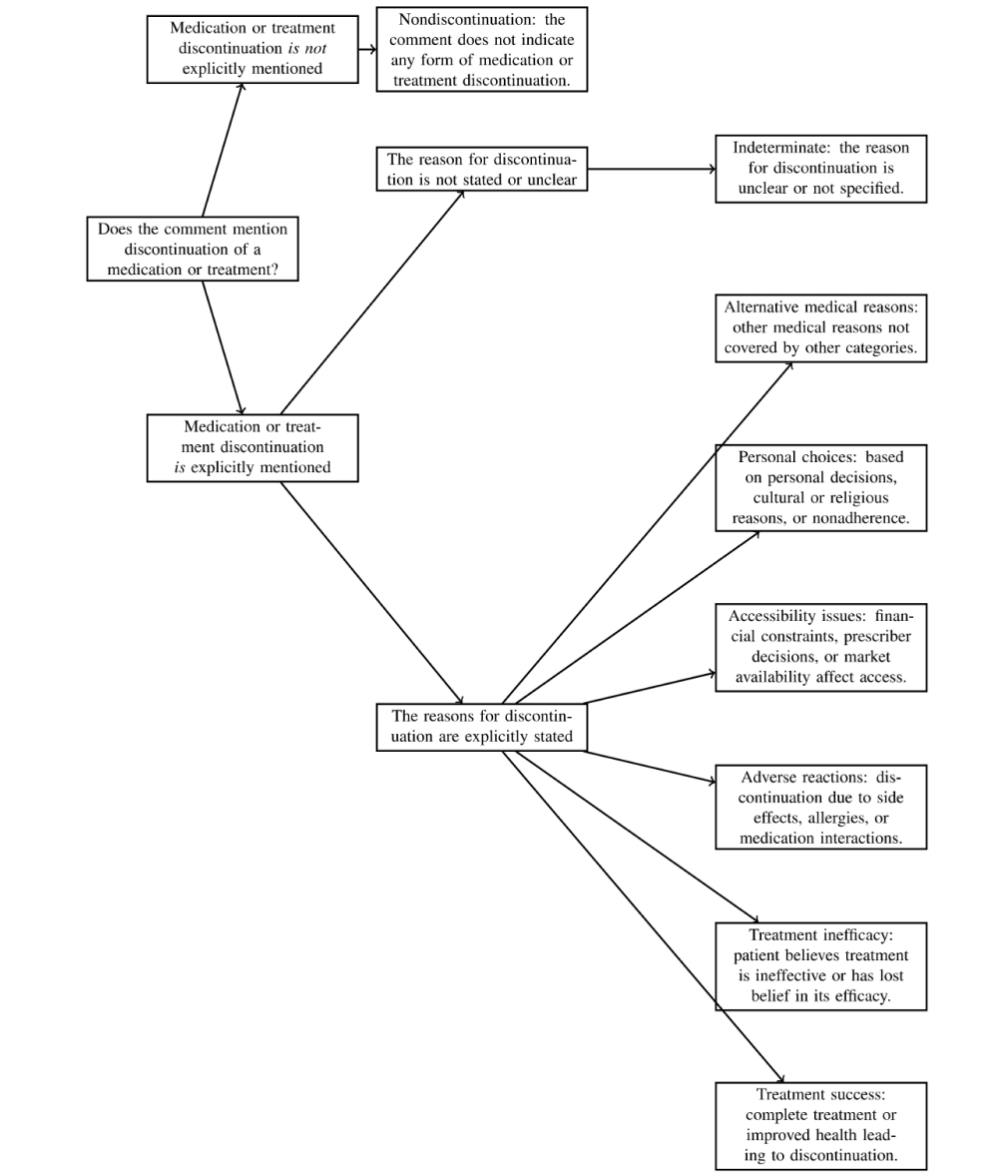
Decision tree for labeling the root causes of drug discontinuation events.

Labelers were instructed to apply these categories to each comment identified as containing a DDE. They could assign multiple categories to a single comment if it mentioned >1 reason for discontinuation. The comprehensive guidelines, including examples and the decision tree shown in Figure 1, helped labelers accurately and consistently classify the root causes of DDEs. The aim was to capture the complex and multifaceted reasons behind medication discontinuation, providing valuable insights for further analysis.

**Categories for root causes.**

- Treatment success: discontinuation due to successful treatment completion or sufficient improvement in health
- Treatment inefficacy: perceived ineffectiveness of the treatment or loss of belief in its efficacy
- Adverse reactions: discontinuation due to adverse side effects, allergic reactions, or negative interactions with other medications
- Accessibility issues: issues related to financial constraints, changes in prescriber decisions, or market availability
- Personal choices: discontinuation based on personal decisions, cultural or religious reasons, or general nonadherence without medical advice
- Alternative medical reasons: other medical reasons not explicitly covered by the other categories
- Indeterminate: unclear or unspecified reasons for discontinuation
- Nondiscontinuation: comments that do not indicate any form of drug discontinuation

### Models

In this study, we used the following models from Hugging Face (Hugging Face, Inc)—all of which are LLMs fine-tuned for zero-shot classification—to detect DDEs in comments posted on web-based forums: nli-deberta-base [31]; bart-large-mnli [32]; roberta-large-mnli [33]; distilbert-base-uncased-mnli [34]; nli-distilroberta-base [35].

**Figure 2:**
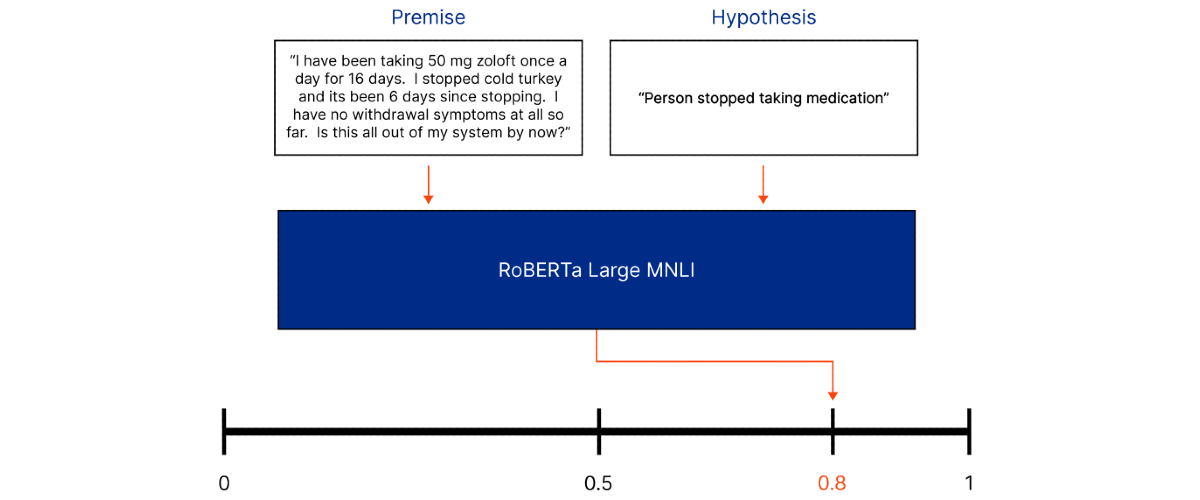
Illustration of how large language models fine-tuned to perform natural language inference can be used to perform zero-shot classification. MNLI: Multi-Genre Natural Language Inference; RoBERTa: Robustly Optimized Bidirectional Encoder Representations from Transformers Pretraining Approach.

In addition, we used OpenAI’s GPT-3.5 Turbo and GPT-4 models [36]. These LLMs have been trained on a wide range of internet text and can generate humanlike text, making them useful for our research. GPT-3.5 Turbo is smaller and less costly than GPT-4 because of its fewer parameters [37]. As illustrated in Figure 2, these models can be used to analyze a premise from a web-based forum post (eg, a post from MedHelp) alongside a hypothesis (eg, “person stopped taking medication”) and output a probability, such as 0.8, for whether the premise entails the hypothesis. This approach allows us to identify potential discontinuation events in a zero-shot manner without requiring additional training data.

### Data Classification Procedure Used to Detect DDEs

Detecting DDEs can be framed as a binary classification task, where the goal is to determine whether a text sequence contains a DDE or not. For example, the statement “patient stopped Lipitor because of cost” would be classified as containing a DDE. Each model, including GPT-3.5 Turbo and GPT-4, performed zero-shot classification as described in the classification strategies later. For models from the Hugging Face library, comments from MedHelp were treated as premises with the hypothesis being the following: “Person stopped taking medication.” Note that the results presented in this paper and the performance of the proposed framework are sensitive to the hypothesis used, and using a different hypothesis could result in different results.

For GPT-3.5 Turbo and GPT-4, the models were prompted to generate a response to a query using the comment as context. The model’s output was processed to derive a probability of entailment, which was used for classification. The evaluation setting for the GPT models varied with the classification strategy used and was influenced by the model version. It is important to note that the DDE detection experiments were performed before the release of GPT-4 Turbo and the release of the “set seed” and structured JavaScript Object Notation response features by OpenAI. As a result, carefully structured prompts were needed to obtain parseable responses from the GPT models for the task of DDE detection. These prompts and the complete code used in this work are available on GitHub [38]. The subsequent sections detail the procedures used to classify comments into the 2 categories, with practical examples of these classification strategies shown as figures.

#### Step 1: Split Each Comment Into Sentences and Tokenize

The models used for DDE detection, including GPT-3.5 Turbo and GPT-4, have a maximum token input length. This means each model can only process text that, when tokenized, is within its maximum token limit. Given that questions and answers on MedHelp can exceed these limits, we split longer comments into smaller chunks for classification. A comment was classified as discussing a DDE if any of its segments were classified as such. To handle the prevalence of typos and missing punctuation, we used the spaCy Python library’s Sentencizer method [39] to segment each question and answer into sentences. Despite some inaccuracies due to informal language, typos, or incorrect punctuation, we referred to the output of the Sentencizer as the consecutive “sentences” forming each question or answer from MedHelp.

#### Step 2: Apply a Classification Strategy

**Figure 3:**
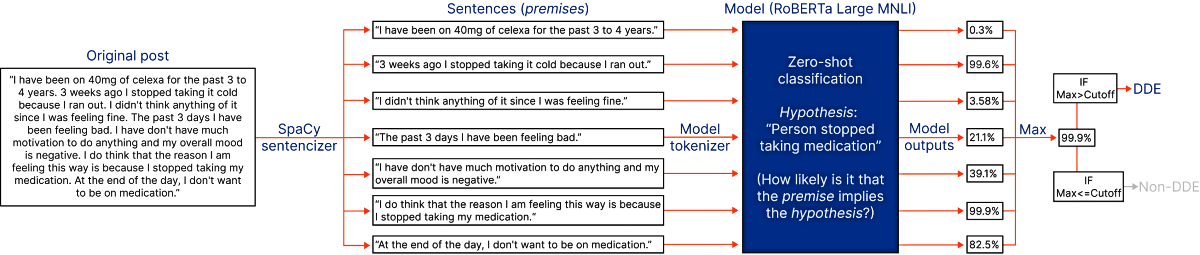
An example illustrating classification strategy 1. DDE: drug discontinuation event; Max: maximum; MNLI: Multi-Genre Natural Language Inference; RoBERTa: Robustly Optimized Bidirectional Encoder Representations from Transformers Pretraining Approach.

**Figure 4:**
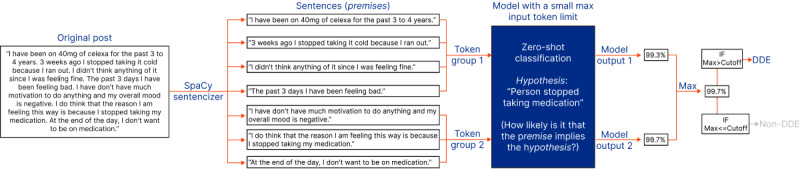
An example illustrating classification strategy 2 when using a model with a large maximum token input length. DDE: drug discontinuation event; Max: maximum; MNLI: Multi-Genre Natural Language Inference; RoBERTa: Robustly Optimized Bidirectional Encoder Representations from Transformers Pretraining Approach.

**Figure 5:**
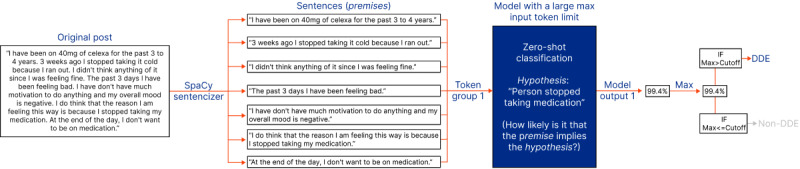
An example illustrating classification strategy 2 when using a model with a small maximum token input length. DDE: drug discontinuation event; Max: maximum; MNLI: Multi-Genre Natural Language Inference; RoBERTa: Robustly Optimized Bidirectional Encoder Representations from Transformers Pretraining Approach.

Each comment was processed using 3 distinct classification strategies: classification strategy 1 (CS1, see Figure 3 and Textbox 2), classification strategy 2 (CS2; see Figures 4 and 5 and Textbox 3), and classification strategy 3 (CS3; see Figure 5 and Textbox 4).

In CS1 (Figure 3; Textbox 2), each sentence from the text is treated as a premise and passed into the model individually. The maximum probability (ie, model output) returned for any premise is identified. If this maximum probability is above a user-defined cutoff level, the original post is classified as containing a DDE. In the example shown in Figure 3, the post is classified as containing a DDE because the maximum model output exceeds the user-defined cutoff level (95%).

In CS2 (Figures 4 and 5; Textbox 3), consecutive sentences are concatenated and passed into the model as single premises. The maximum model output for any premise is compared to a user-defined cutoff value. In the example shown in Figure 4, the post is classified as containing a DDE because the maximum probability (99.4%) is higher than the cutoff level (95%).

In Figure 5, an example shows a scenario where the total number of tokens in the original post exceeds the maximum input length that the model can accept. Here, sequential sentences are grouped to ensure no group exceeds the model’s maximum token limit (Textbox 4).

**CS1—classification strategy 1: individual sentences.**

1. Each sentence in a comment was classified individually, generating a “model output” for each sentence ranging from 0 (non-DDE) to 1 (DDE).
2. The “model prediction” was set as the maximum of all the “model outputs” for the sentences.
3. If the “model prediction” exceeded a user-defined cutoff value, the comment was classified as a DDE. Otherwise, it was categorized as a non-DDE.

**CS2—classification strategy 2: groups of sentences.**

1. Groups of sentences were created by concatenating as many consecutive sentences together as possible while staying within the maximum token input length of the model.
2. Each group of sentences was classified as a single block of text, generating a “model output” for each group of sentences ranging from 0 (non-DDE) to 1 (DDE).
3. The “model prediction” was set as the maximum of all the “model outputs” for the different groups of sentences.
4. If the “model prediction” exceeded a user-defined cutoff value, the comment was classified as a DDE. Otherwise, it was classified as a non-DDE.

**CS3—classification strategy 3: entire text (ChatGPT only).**

The entire text is passed into the ChatGPT model at once, and the model is prompted to provide a binary “1” (DDE) or “0” (non-DDE) classification for each comment.

#### Step 3: Compare the “Model Prediction” to a User-Defined “Cutoff” Value

To derive a binary classification for a comment from the set of model outputs for each sentence (CS1) or block of sentences (CS2), we compared the maximum of all the model outputs to a cutoff value. If the maximum model output for any sentence or block of sentences is greater than the cutoff value, the comment is classified as a DDE. Otherwise, it is classified as a non-DDE. Cutoff values were tested from 0.05 to 0.95 in increments of 0.05. Adjusting the cutoff impacts the trade-off between sensitivity and specificity, reflected in the resulting false positive rate (FPR) and false negative rate (FNR) of each classifier. This approach helps identify an optimal cutoff that balances false positives and false negatives. This structured approach ensures a comprehensive analysis and maximizes the accuracy of DDE detection in unstructured web-based forum data.

### Data Classification Procedure Used to Detect the Root Causes of DDEs

Once DDEs were identified, we proceeded to classify the root causes of these discontinuations. The root cause classification was treated as a multilabel classification task, allowing each comment to be associated with multiple reasons for discontinuation.

#### Applying CS1, CS2, and CS3 to DDE Root Cause Classification

The same classification strategies (ie, CS1, CS2, and CS3) used to detect DDEs were applied to identify the root causes of DDEs.

##### CS1: Individual Sentences

Each sentence in a DDE-identified comment was classified individually to determine the root causes. Each sentence was analyzed to see if it fit any of the predefined root cause categories in the Classifying the Root Causes of DDEs section.

If any sentence indicated a root cause, it was considered a relevant cause for the entire comment. The final set of root causes for the comment was derived from the union of causes identified in individual sentences.

##### CS2: Groups of Sentences

Groups of sentences were classified together to determine the root causes. This method ensured that contextual information was preserved, which is crucial for accurately identifying root causes that may span multiple sentences. Each group of sentences was evaluated against the predefined categories of root causes, and the maximum model output for each group determined the presence of specific root causes.

##### CS3: Entire Comment

The entire text of each DDE-identified comment was passed into the ChatGPT model to classify the root causes. The model was prompted to first summarize the reasons for discontinuation and then was asked to generate a list of applicable root causes from the predefined categories. This approach leveraged the model’s ability to understand the context of the entire comment, providing a comprehensive assessment of the root causes.

#### Comparing Model Predictions to Cutoff Values

For both CS1 and CS2, the maximum model output for each root cause category was compared to a user-defined cutoff value. If the model output exceeded the cutoff, the root cause was assigned to the comment.

The complete set of prompts and hypotheses used for both DDE detection and root cause analysis can be found on our GitHub repository [38]. These details are omitted here for brevity, but they are available on the web and in Multimedia Appendix 1 to ensure transparency and facilitate replication of our methods. Researchers and practitioners can access these resources to better understand the specific inputs used in our classification models and apply similar techniques to their own datasets.

### Ethical Considerations

This study involves the analysis of user-generated content on MedHelp, a publicly accessible health forum where individuals share personal medical experiences and seek health advice. Given the sensitive nature of the health information discussed, we are committed to protecting user privacy and ensuring ethical handling of all data. While the content on MedHelp is publicly available, users have not explicitly consented to participate in this research. We acknowledged the need to respect users’ implied expectations of privacy, especially given the health-focused context of the discussions. No attempt was made to contact individual users nor were they asked to participate directly in this study. The scraped data have been processed in a way that prevents the association of responses or health conditions with specific users. This study complies with all relevant ethical guidelines for internet-based research, and we did not find it necessary to seek an ethics review board assessment based on the guidelines set by the University of Florida institutional review board.

## Results

### Overview

This section presents the results of our experiments on detecting DDEs and identifying their root causes. We evaluated the performance of various LLMs, using different classification strategies. The evaluation included precision-recall (PR) curves, receiver operating characteristic (ROC) curves, and detailed performance metrics for each classifier and strategy combination.

### DDE Detection Task

**Figure 6:**
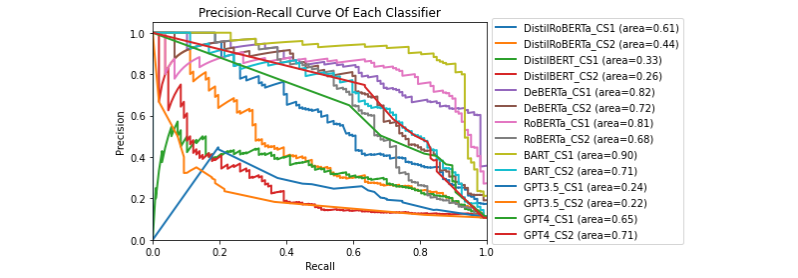
The precision-recall curve of each classifier on the task of detecting drug discontinuation events. BART: Bidirectional and Auto-Regressive Transformers; DeBERTa: Decoding-Enhanced Bidirectional Encoder Representations from Transformers with Disentangled Attention; RoBERTa: Robustly Optimized Bidirectional Encoder Representations from Transformers Pretraining Approach.

**Figure 7:**
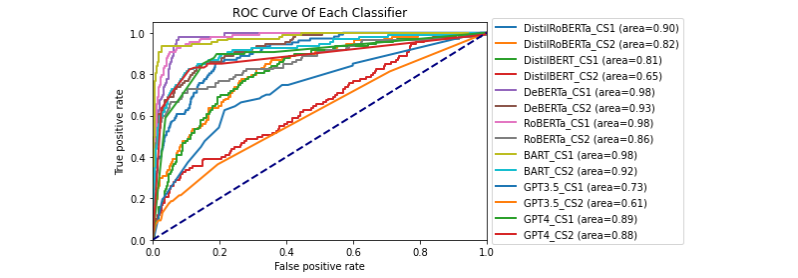
The receiver operating characteristic curve of each classifier on the task of detecting drug discontinuation events. BART: Bidirectional and Auto-Regressive Transformers; DeBERTa: Decoding-Enhanced Bidirectional Encoder Representations from Transformers with Disentangled Attention; RoBERTa: Robustly Optimized Bidirectional Encoder Representations from Transformers Pretraining Approach.

The effectiveness of the classifiers in detecting DDEs was evaluated using PR and ROC curves. Figures 6 and 7 show the PR and ROC curves for each classifier under each classification strategy, respectively. These figures also include the area under the curve (AUC) for each combination of classifier and strategy. Of all the models and classification strategies, bidirectional and auto-regressive transformers (BART) under CS1 achieved the highest ROC AUC of 0.977 and the highest PR AUC of 0.904. On the other hand, GPT-3.5 Turbo under CS2 had the lowest ROC AUC of 0.611 and the lowest PR AUC of 0.215.

Under CS3, GPT-4 achieved an *F*_1_-score of only 0.56, an FPR of 12.9%, an FNR of 18.7%, and an accuracy of 86.5%. GPT-3.5 Turbo performed even more poorly under CS3, achieving an *F*_1_-score of only 0.27, an FPR of 5.6%, an FNR of 76.6%, and an accuracy of 86.8%, illustrating the imbalance in the dataset. In comparison, the best-performing classifier at the task of DDE detection was BART under CS1, achieving an *F*_1_-score of 0.86, an accuracy of 96.8%, an FPR of 2.8%, and an FNR of 6.5%. A summary of these performance metrics can be found in Tables S1-S9 in Multimedia Appendix 1.

### DDE Root Cause Classification Task

To assess the performance of the classifiers in identifying the root causes of DDEs, we used multiple evaluation metrics, including micro-average ROC AUC, macro-average ROC AUC, weighted-average ROC AUC, micro-average PR AUC, macro-average PR AUC, weighted-average. PR AUC, micro-average *F_1_*, macro-average *F*_1_, weighted-average *F*_1_, hamming loss, Jaccard score, and subset accuracy. The results across all DDE root causes are summarized in Tables 1 and 2.

Tables 3 and 4 show the performance of GPT-4 Turbo and GPT-4o at predicting each individual DDE root cause. Overall, GPT-4o performed the best across the board achieving the highest averaged *F*_1_-scores, the lowest hamming loss, and the highest Jaccard score and subset accuracy of all the classifiers. On the other hand, BART and DeBERTa performed very poorly across the board on the task of DDE root cause detection as shown in Tables 1 and 2.

An overview of the frequency of each DDE root cause in our labeled dataset of 1000 comments is provided in Table 5.

The most common DDE root causes in our dataset were *personal choices* with 670 (67%) occurrences and *adverse reactions* with 627 (62.7%) occurrences, and the least common was *treatment success* with 96 (9.6%) occurrences. In addition, only 10% (100/1000) of the comments were labeled as non-DDE by labelers, suggesting the accuracy performance is consistent with the experiments performed regarding DDE detection in a smaller labeled dataset.

**Table 1:** Evaluation metrics for DDE^a^ root cause classification (part 1).

Classifier	Strategy	Micro-average ROC^b^ AUC^c^	Macro-average ROC AUC	Weighted-average ROC AUC	Micro-average PR^d^ AUC	Macro-average PR AUC	Weighted-average PR AUC
DeBERTa^e^	CS1^f^	0.568669	0.507989	0.502451	0.338243	0.300314	0.472326
DeBERTa	CS2^g^	0.528049	0.508299	0.500792	0.312381	0.300947	0.472003
BART^h^	CS1	0.604359	0.510527	0.507081	0.368773	0.301987	0.474543
BART	CS2	0.581217	0.518657	0.518826	0.344869	0.306675	0.480845

^a^DDE: drug discontinuation event.

^b^ROC: receiver operating characteristic.

^c^AUC: area under the curve.

^d^PR: precision-recall.

^e^DeBERTa: Decoding-Enhanced Bidirectional Encoder Representations from Transformer with Disentangled Attention.

^f^CS1: classification strategy 1.

^g^CS2: classification strategy 2.

^h^BART: Bidirectional and Auto-Regressive Transformers.

**Table 2:** Evaluation metrics for DDE^a^ root cause classification (part 2).

Classifier	Strategy	Micro-average *F*_*1*_	Macro-average *F*_*1*_	Weighted-average *F*_*1*_	Hamming loss	Jaccard score	Subset accuracy
DeBERTa^b^	CS1^c^	0.337991	0.245975	0.359274	0.36594	0.218692	0.014905
DeBERTa	CS2^d^	0.222708	0.184899	0.244992	0.366095	0.119609	0.005095
BART^e^	CS1	0.425243	0.284461	0.441498	0.363548	0.292373	0.029905
BART	CS2	0.445648	0.349929	0.516783	0.469571	0.297445	0.015476
GPT-4 Turbo	CS3^f^	0.729443	0.616037	0.711924	0.153	0.634567	0.344
GPT-4o	CS3	0.783084	0.739096	0.786899	0.128875	0.6823	0.356

^a^DDE: drug discontinuation event.

^b^DEBRTa: Decoding-Enhanced Bidirectional Encoder Representations from Transformer with Disentangled Attention.

^c^CS1: classification strategy 1.

^d^CS2: classification strategy 2.

^e^BART: Bidirectional and Auto-Regressive Transformers.

^f^CS3: classification strategy 3.

**Table 3:** Detailed performance metrics by DDE^a^ root cause for GPT-4 Turbo.

Class	*F*_1_-score	Precision	Recall	Accuracy	FPR^b^	TPR^c^	FNR^d^	TNR^e^
Treatment success	0.6339	0.6667	0.6042	0.933	0.0321	0.6042	0.3958	0.9679
Treatment inefficacy	0.6840	0.6840	0.6840	0.842	0.1053	0.6840	0.3160	0.8947
Adverse reactions	0.8467	0.8267	0.8676	0.803	0.3056	0.8676	0.1324	0.6944
Accessibility issues	0.6524	0.8971	0.5126	0.935	0.0079	0.5126	0.4874	0.9921
Personal choices	0.8138	0.8362	0.7925	0.757	0.3152	0.7925	0.2075	0.6848
Alternative medical reasons	0.5690	0.6918	0.4833	0.694	0.1546	0.4833	0.5167	0.8454
Indeterminate	0.0702	0.3636	0.0388	0.894	0.0078	0.0388	0.9612	0.9922
Non-DDE	0.6583	0.5643	0.7900	0.918	0.0678	0.7900	0.2100	0.9322

^a^DDE: drug discontinuation event.

^b^FPR: false positive rate.

^c^TPR: true positive rate.

^d^FNR: false negative rate.

^e^TNR: true negative rate.

**Table 4:** Detailed performance metrics by DDE^a^ root cause for GPT-4o.

Class	*F*_1_-score	Precision	Recall	Accuracy	FPR^b^	TPR^c^	FNR^d^	TNR^e^
Treatment success	0.7551	0.74	0.7708	0.952	0.028	0.7708	0.2292	0.9712
Treatment inefficacy	0.7968	0.8016	0.792	0.899	0.0653	0.792	0.208	0.9347
Adverse reactions	0.8836	0.9089	0.8596	0.858	0.1448	0.8596	0.1404	0.8552
Accessibility issues	0.8087	0.8378	0.7815	0.956	0.0204	0.7815	0.2185	0.9796
Personal choices	0.8419	0.8576	0.8269	0.792	0.2788	0.8269	0.1731	0.7212
Alternative medical reasons	0.6457	0.6295	0.6627	0.696	0.2801	0.6627	0.3373	0.7199
Indeterminate	0.3810	0.3438	0.4272	0.857	0.0937	0.4272	0.5728	0.9064
Non-DDE	0.8000	0.7810	0.8200	0.959	0.0256	0.8200	0.1800	0.9744

^a^DDE: drug discontinuation event.

^b^FPR: false positive rate.

^c^TPR: true positive rate.

^d^FNR: false negative rate.

^e^TNR: true negative rate.

**Table 5:** Frequency of labels in the dataset.

Label	Comments (n=1000), n (%)
Personal choices	670 (67)
Adverse reactions	627 (62.7)
Alternative medical reasons	418 (41.8)
Treatment inefficacy	250 (25)
Accessibility issues	119 (11.9)
Indeterminate	103 (10.3)
Nondiscontinuation	100 (10)
Treatment success	96 (9.6)

## Discussion

### DDE Detection

#### Overview

The results from the PR and ROC curves highlight significant differences in the performance of the models at detecting DDEs using the 3 different classification strategies: CS1, CS2, and CS3. These metrics are essential for understanding the trade-offs between sensitivity and specificity in identifying DDEs, which is vital in clinical settings where missing a DDE can have serious implications.

The PR curves (Figure 6) demonstrate the effectiveness of each classifier in handling the imbalanced nature of the dataset. BART under CS1 achieved the highest PR AUC, indicating its superior ability to maintain high precision without sacrificing recall. This performance suggests that BART is particularly adept at identifying DDEs in a dataset where DDEs are relatively rare, representing only 107 (10.7%) of the 1000 comments we labeled. In contrast, GPT-3.5 Turbo under CS2 had the lowest *F*_1_-score of only 0.22, showing significant challenges in balancing precision and recall. These results highlight the limitations of some models when applied with the classification strategies we developed and to our imbalanced data set. However, these results highly depend on the prompts used with the models, and better results may be achieved by using different prompts in conjunction with the proposed classification strategies. Further research into this area is needed to better understand this phenomenon.

Similarly, the ROC curves (Figure 7) illustrate the classifiers’ ability to distinguish between DDE and non-DDE cases across various thresholds. The area under the ROC curve provides a measure of the overall discriminative power of the models. BART’s high area under the ROC curve under CS1 indicates its robustness in differentiating between the 2 classes, even at different decision thresholds. This robustness is crucial in clinical applications where both high sensitivity and specificity are required to minimize false negatives and false positives.

#### Classification Strategies

##### CS1: Individual Sentences

This strategy was particularly effective for both DeBERTa and BART in detecting DDEs, maintaining a higher balance between precision and recall as evidenced by their PR AUC scores. By analyzing sentences individually, this approach mitigates the dilution of relevant information, which is particularly beneficial in contexts where critical information might be embedded within longer text.

##### CS2: Groups of Sentences

While this strategy provided context by combining multiple sentences, it often resulted in a higher FNR due to the dilution of relevant information with surrounding text. This observation aligns with our hypothesis that feeding multiple sentences to a model simultaneously can lead to relevant DDE mentions being overshadowed by unrelated information. However, an intriguing exception was observed for GPT-4, which performed slightly better under CS2 than CS1 at higher cutoff values. This suggests that GPT-4 might better leverage the entire context of comments to make more informed classifications, indicating its potential to handle more complex and detailed contexts.

##### CS3: Entire Comment (GPT 3.5 Turbo and GPT-4 Models Only)

Interestingly, the performance of GPT-4 under CS3 was comparable to its performance under CS2 with a cutoff of 0.75, contradicting our initial hypothesis that the GPT models would perform better under the binary classification task considered in CS3. This implies that the architecture and training methodology of GPT-4 may not align with the approach implemented in CS3 or that our prompt design in CS3 was not optimally formulated. Further investigation is needed to understand this phenomenon better.

#### Key Takeaways

##### Superior Performance of BART

BART’s superior performance, particularly under CS1, highlights its robustness and reliability in detecting DDEs from unstructured patient-generated content. This finding suggests that BART can be effectively used in clinical research to identify relevant data from imbalanced datasets without the need for extensive fine-tuning, which is crucial for real-world applications where data imbalance is common.

Because of BART’s exceptional performance in DDE detection, we used BART under the classification strategy and cutoff that it achieved the best performance with to curate the dataset we labeled and used for our DDE root cause analysis. BART achieved the highest *F*_1_-score of 0.86 under CS1 with a cutoff of 0.9.

##### Class Imbalance

The significant class imbalance, with only 107 out of 1000 (10.7%) comments labeled as DDEs, emphasizes the need for careful evaluation of classifier performance beyond overall accuracy. Metrics such as PR AUC provide a more comprehensive understanding of model performance, ensuring that high accuracy does not mask poor performance in detecting less frequent but critical events.

##### Real-World Applications

High FPRs and FNRs in DDE detection can lead to missed DDEs or unnecessary interventions, respectively. Therefore, achieving a balance in FPR and FNR, as demonstrated by BART under CS1 with a cutoff of 0.9, is essential for practical applications. This balance ensures that models can effectively support health care providers in making informed decisions based on patient-generated data.

### DDE Root Cause Analysis

Our multilabel classification approach enabled the identification of multiple reasons for drug discontinuation within a single comment, offering a comprehensive understanding of patient behavior and decision-making. This robust methodology allowed us to leverage the strengths of LLMs to tackle complex clinical research questions related to DDEs.

### Performance Highlights

#### DeBERTa and BART Struggled at Detecting DDE Root Causes

Both models struggled significantly at identifying the root causes of DDEs, as indicated by their much lower *F*_1_-scores and higher hamming loss in Table 2 in comparison to GPT-4 Turbo and GPT-4o. Another interesting observation was that 10% (100/1000) of the comments in the dataset were non-DDEs, meaning that BART under CS1 with a cutoff of 0.9 inaccurately classified them as DDEs. We noticed that BART tended to label non-DDE comments as DDEs if they contained phrases such as “I am not on any medication.” This is an important takeaway as it shows that even the models that performed the best at detecting DDEs still have tendencies to make mistakes in certain scenarios.

#### GPT-4 Turbo and GPT-4o Performed Exceptionally Well at Detecting DDE Root Causes

These models outperformed DeBERTa and BART across most metrics. GPT-4o, in particular, achieved the highest *F*_1_-scores, lowest hamming loss, and highest Jaccard scores, indicating its superior ability to accurately and reliably classify the root causes of DDEs. The advanced capabilities of GPT-4 Turbo and GPT-4o in processing complex textual inputs holistically contributed to their outstanding performance.

#### Classification Strategies

##### CS1 (Individual Sentences) and CS2 (Groups of Sentences)

Although these strategies were effective for both DeBERTa and BART at detecting DDEs, the models struggled at detecting the root causes of DDEs under these strategies, as indicated by their extremely poor performance results shown in Tables 1 and 2.

##### CS3: Entire Comment (GPT-4 Turbo and GPT-4o Models Only)

GPT-4 Turbo and GPT-4o performed exceptionally well under CS3, likely due to their advanced capabilities in understanding and processing complex textual inputs holistically. This strategy allowed the models to capture the full context of the comments, leading to more accurate and reliable DDE root cause identification.

#### Key Takeaways

##### Superior Performance of GPT-4 Turbo and GPT-4o

The outstanding performance of GPT-4 Turbo and GPT-4o models highlights the importance of using advanced language models for complex classification tasks in health care. Their ability to accurately identify the root causes of DDEs can provide valuable insights for health care providers, enabling them to address patient concerns more effectively and improve medication adherence.

##### Challenges With Indeterminate Class

One notable challenge was the performance of GPT-4 Turbo in detecting DDEs belonging to the *indeterminate* class. For instance, a comment such as “My dad was diagnosed with bipolar disorder and stopped taking his meds after being in a rehabilitation center twice” did not clearly state the reason for discontinuation. Instead of classifying this as *indeterminate*, GPT-4 Turbo attempted to infer specific reasons such as *personal choices* or *adverse reactions.* This tendency to infer explicit conditions from ambiguous statements indicates a potential overfitting issue, where the model predicts more common classes instead of recognizing the ambiguity inherent in the input.

##### Class Imbalance

The significant class imbalance presented a challenge for all classifiers. Classes with higher frequencies, such as *personal choices* and *adverse reactions*, exhibited better performance. In contrast, less frequent classes such as *indeterminate* showed lower performance, highlighting the need for more balanced training datasets or advanced techniques to handle class imbalance effectively.

### Clinical Implications

The superior performance of GPT-4 Turbo and GPT-4o in both DDE detection and root cause analysis underscores their potential application in clinical settings. These models can help health care providers identify common reasons for drug discontinuation, enabling the development of targeted interventions to improve medication adherence and patient outcomes. By leveraging these advanced NLP models, clinicians and researchers can gain deeper insights into patient behavior and decision-making processes, facilitating more effective and personalized health care strategies.

This research emphasizes the importance of capturing the patient’s perspective directly from web-based health forums, which offers a richer and more authentic understanding of patient behavior and the real-world challenges they face compared to traditional electronic health record data. By identifying the root causes of DDEs from the patient’s standpoint, health care providers, policy makers, and pharmaceutical companies can develop more targeted interventions to enhance medication adherence and patient outcomes.

### Future Research Directions

To further enhance the applicability and reliability of these models in real-world settings, future research should focus on the opportunities outlined in the subsequent sections.

#### Real-World Applications

This research provides a foundation for clinical applications, such as improving patient adherence, by identifying common reasons for drug discontinuation. Health care providers can use these insights to develop targeted interventions and support systems to address the specific challenges faced by patients, ultimately enhancing treatment outcomes. Furthermore, applying these findings in real-world settings, such as clinical decision support systems, where accurate and reliable classification of text can significantly impact patient outcomes and health care processes, represents a substantial opportunity. Developing scalable and cost-effective solutions for deploying these models in practice will be crucial.

#### Prompt Optimization

Future research should explore various prompt optimization techniques to enhance model performance. This includes experimenting with chain-of-thought prompting, which has been shown to improve the performance of LLMs on complex reasoning tasks. In addition, investigating the impact of prompt specificity and structure on DDE root cause classification performance can provide valuable insights into optimal prompt design. For instance, future research could examine how the performance of GPT-4 Turbo and GPT-4o changes when examples of positive instances for low-frequency classes are included in the prompt. One concern with this approach is the potential for “prompt dependency,” where providing specific examples might restrict the model’s ability to generalize and accurately classify comments that differ in nature from those presented in the prompt.

#### Exploring Other State-of-the-Art LLMs

Research should be expanded to include state-of-the-art language models from developers other than OpenAI, such as models from Anthropic, Google, and Cohere. Models such as Claude by Anthropic and Gemini by Google have shown promising results in various NLP tasks and could be valuable for this study. In addition, models from research institutions, such as Command R by Cohere, should also be considered for comprehensive performance comparisons. By comparing the performance of these models on DDE root cause classification, we can identify the best tools for specific multilabel classification tasks and potentially reduce dependency on any single provider. This comparative analysis will help in understanding the strengths and limitations of each model in handling complex health care–related data, ultimately leading to more robust and versatile NLP applications.

#### Cost Minimization Strategies

Developing strategies to minimize the costs associated with using advanced NLP models is essential. This includes exploring open-source alternatives, such as LLaMA, optimizing model use to reduce application programming interface calls, and implementing efficient data processing pipelines. Research directions toward cost minimization could potentially align with advanced prompting techniques, such as classifying multiple comments with a single prompt and application programming interface call. However, preliminary research suggests that longer prompts lead to degraded performance of LLMs, even when the number of tasks in a single prompt does not increase.

### Limitations

In summary, this research provides valuable insights into the ability of LLMs to detect DDEs from textual comments. However, the results should be interpreted with some caution due to the small sample size of DDE-labeled comments and the case imbalance inherent in our dataset. It is also worth mentioning that, as our study was restricted to English language comments from MedHelp, the generalizability of our results to other languages and platforms is not guaranteed. Furthermore, because the models used in this work generate stochastic outputs, the reproducibility of these results may be limited.

Despite these limitations, our study provides a foundation for further exploration into the use of LLMs in health informatics, specifically in identifying DDEs. Future studies should focus on improving the performance of transformer models by investigating other strategies, exploring ensemble methods, refining the classification prompt for the ChatGPT models, or incorporating different forms of data augmentation.

We anticipate that such innovative methods will stimulate and support further research into drug discontinuation, aiding health care researchers in identifying factors associated with the discontinuation of specific medications and treatments. These advancements are crucial in spotlighting new research directions with the potential to ameliorate treatment protocols and mitigate issues associated with medication discontinuation and nonadherence.

The ultimate aim is to enhance the capability of these models in real-world applications where accurate, timely detection of DDEs is of paramount importance. Improved detection can contribute to patient safety, optimize drug therapies, and facilitate better health outcomes.

### Conclusions

This study has provided a thorough evaluation of advanced LLMs for detecting DDEs and identifying their root causes using data from MedHelp. The research demonstrates the effectiveness of state-of-the-art LLM models, particularly GPT-4 and GPT-4o, in handling complex multilabel classification tasks without the need for extensive task-specific training.

In conclusion, this research contributes significantly to the field of NLP and health care informatics by showcasing the potential of advanced classifiers to derive actionable insights from patient-generated content. The findings underscore the transformative impact these technologies can have on understanding and improving medication adherence, ultimately leading to better health care outcomes.

## Appendix

Multimedia Appendix 1 Additional details regarding the methods and results presented in this research.

## Abbreviations

AI: artificial intelligence
AUC: area under the curve
BART: Bidirectional and Auto-Regressive Transformers
CS1: classification strategy 1
CS2: classification strategy 2
CS3: classification strategy 3
DDE: drug discontinuation event
DeBERTa: Decoding-Enhanced Bidirectional Encoder Representations from Transformers with Disentangled Attention
FNR: false negative rate
FPR: false positive rate
LLM: large language model
NLP: natural language processing
PR: precision-recall
ROC: receiver operating characteristic

## Data Availability

The datasets generated and analyzed during this study are openly available in the GitHub repository [38]. This repository includes the raw data, processed data, and any scripts used in data processing and analysis. Further documentation is provided within the repository to guide data use and interpretation. For any additional inquiries regarding the dataset, please contact the corresponding author.
