# Supplementary material for: Using Large Language Models to Detect and Understand Drug Discontinuation Events in Web-Based Forums: Development and Validation Study
Source: J Med Internet Res. 2025 Jan 30;27:e54601. doi: 10.2196/54601 (PMC11826943; doi:10.2196/54601)
Supplement: Multimedia Appendix 1 [file jmir_v27i1e54601_app1.docx]

## Additional Information on Methods

### Motivation behind CS1, CS2, and CS3

The maximum token input length for NLI-DeBERTa-Base and DistilBERT-Base-Uncased-MNLI is 512 tokens, for RoBERTa-Large-MNLI and NLI-DistilRoBERTa-Base it is 514 tokens, for BART-Large-MNLI it is 1024 tokens, for GPT-3.5 Turbo it is 4096 tokens, and for GPT-4 it is 8192. It is worth noting that an even larger GPT-4 model exists, GPT-4-32k, which can support a context up to 32768 tokens.

#### Motivation behind CS1

In CS1, by classifying each sentence individually, we hypothesized that the models may be able to more effectively classify questions or answers which are long, but which only briefly mention a DDE. In such a case, we hypothesized that if a long question or answer largely talked about things not related to a DDE but briefly mentioned a DDE somewhere in the text, the models may have a hard time classifying such questions or answers as DDEs due to the “noise” throughout the text. By running the model on each individual sentence in a comment, then if any sentence discusses a DDE, other irrelevant sentences will not hinder the ability of the models to detect the DDE as the maximum model output for any sentence is taken to be the “model prediction” under CS1. However, by passing each sentence into the model individually, each sentence is taken out of context of the larger comment. Therefore, if a question or answer describes a DDE in an indirect way throughout many sentences, using CS1, the models may not be able to detect the DDE since each sentence is taken out of context of the larger comment. An example of how this strategy is implemented is shown in Figure 3. In the computation results in Section 2, Columns with a (1) such as “DeBERTa (1)” indicate that CS1 was utilized.

#### Motivation behind CS2

Next, to address situations where a DDE may be described in an indirect way throughout many sentences, we developed CS2 to explore if some models might perform better if they had access to the entire context of the comment at once, or at least multiple consecutive sentences concatenated together. In this way, we developed CS2 to evaluate how well the models would perform if we passed in multiple sentences (potentially the entire comment) as single blocks of text. For each model we broke each comment into groups of consecutive sentences which were as large as possible while staying under the maximum token input length of the model. For example, consider a question / answer comprised of five sentences, each of which is 250 tokens in length when tokenized by DistilBERT-Base-Uncased-MNLI’s tokenizer. Since DistilBERT-Base-Uncased-MNLI’s max in- put token length is 512 tokens, we would group the five sentences into three groups. Group one would consist of the first and second sentences (500 tokens in length), group two would consist of the third and fourth sentences (500 tokens in length), and group five would consist of the fifth sentence (250 tokens in length). Here, we have three groups, each of which is less than the max number of tokens for the model, and the first two are as large as they could be since adding the next consecutive sentence to the group would cause the total length to exceed the 512 token limit. In our method, the groups are defined one at a time (group one is formed, then group two, then group three, etc.), and each group will be comprised of as many consecutive sentences as possible while maintaining a total token length less than the maximum length for the model. More examples showing how this strategy is implemented are shown in Figures 5 and 6. In the computation results in Section 2, Columns with a (2) such as “DeBERTa (2)” indicate that CS2 was utilized.

It is worth noting that the maximum token input length varies significantly across the models we tested, ranging from 512 tokens to 8192 tokens. Specifically, the maximum token input length for NLI-DeBERTa-Base and DistilBERT-Base-Uncased-MNLI is 512 tokens, for RoBERTa-Large- MNLI and NLI-DistilRoBERTa-Base it is 514 tokens, for BART-Large-MNLI it is 1024 tokens, for GPT-3.5 Turbo it is 4096 tokens, and for GPT-4 it is 8192. Although the base GPT-4 model offered by OpenAI was sufficient for our research, an even larger GPT-4 model is also offered by OpenAI, GPT-4-32k, which can support a context up to 32768 tokens [43].

#### Motivation behind CS3

While the HuggingFace models naturally output a probability of entailment for each Premise, carefully curated prompts were designed for the ChatGPT models to reliably obtain a structured response which could be parsed to obtain a probability of entailment to allow for a direct com- parison with the HuggingFace models using CS1 and CS2. Furthermore, since ChatGPT does not naturally output a probability of entailment like the HuggingFace models do, we hypothesized that asking the model to give a probability of entailment might not align well with its inherent text generation functionality. Instead, we hypothesized that a binary classification task where the model is explicitly asked to predict whether a text does or does not describe a DDE could be a better fit for the capabilities of ChatGPT, and potentially yield better performance. As a result, we developed CS3 to test this hypothesis on the GPT-3.5 Turbo and GPT-4 models specifically.

### ChatGPT prompt design for CS1, CS2, and CS3

GPT models including GPT-3.5- Turbo and GPT-4 are sequence-to-sequence models meaning that the output is narrative, and as a result, prompts for GPT models must be carefully curated in order to obtain results in consistent formats.

#### ChatGPT prompt used in CS1 and CS2 for DDE Detection

Your task as an AI model is to determine the probability that a given comment from medhelp.org entails a “Drug Discontinuation Event”.

The concept of a “Drug Discontinuation Event” involves a specific individual stopping a recurring medication or treatment. It includes instances where the individual has switched from one medication to another, but not one-time treatments.

To formulate your task, consider each comment as a premise and the statement “A person stopped taking a medication” as the hypothesis. Your goal is to estimate the probability that the hypothesis is true given the premise. Express this probability as a percentage.

For example, if a comment strongly implies a Drug Discontinuation Event, you might respond with “Probability of entailment: 95%”. Conversely, if a comment does not suggest a Drug Discontinuation Event, you might respond with “Probability of entailment: 5%”.

Examples:

1. Comment: “I stopped taking my birth control medication.” Response: “Probability of entailment: 100%”
2. Comment: “I took the plan B pill yesterday.” Response: “Probability of entailment: 5%”
3. Comment: “I changed my birth control medication because of side effects.” Response: “Probability of entailment: 95%”
4. Comment: “I stopped taking my birth control medication because I was feeling worse, but then I started taking it again.” Response: “Probability of entailment: 100%”

Next, I will provide a comment, and you will estimate the probability of entailment as in- structed. Remember, the output should be in the format of “Probability of entailment: X%” where X is the estimated probability.

#### ChatGPT prompt used in CS3 for DDE Detection

As an AI model, your task is to classify each comment from medhelp.org into one of two categories based on the content of the comment. The categories are: “Drug Discontinuation Event” (1) and “Non-Drug Discontinuation Event” (0).

A “Drug Discontinuation Event” (1) is any instance where it can be deduced from the comment that a specific individual has stopped a recurring medication or treatment. This includes cases where the individual has switched from one medication to another. It does not include one-time treatments.

A “Non-Drug Discontinuation Event” (0) is any instance where it cannot be inferred from the comment that a specific individual has stopped a recurring medication or treatment.

Examples:

1. Comment: “I stopped taking my birth control medication” Response: “1”
2. Comment: “I took the plan B pill yesterday” Response: “0”
3. Comment: “I changed my birth control medication because of side effects” Response: “1”
4. Comment: “I stopped taking my birth control medication because I was feeling worse, but then I started taking it again” Response: “1”

Next, I will give you a comment, and you will classify it according to these instructions. Re- member to only respond with “1” or “0”.

#### ChatGPT prompt used for DDE Root-Cause Detection

Below are the system and user prompts utilized with GPT-4 and GPT-4o for DDE root-cause detection.

System Prompt

"Function as the leading expert on patient narratives from MedHelp.org, with a profound understanding of medication and treatment discontinuation reasons discussed on the forum. Utilize your comprehensive knowledge of health issues, treatments, patient concerns, and the typical language, including acronyms and slang used on MedHelp.org. Your expertise encompasses interpreting comments related to changes in patient behavior regarding medical treatments or medication, including reasons for discontinuation or adherence challenges."

User Prompt

“””

Analyze the following comment from MedHelp.org and provide responses in structured JSON format:

1. Describe the rationale behind the author's decision to discontinue their medication or treatment as depicted in the comment.

2. Identify all applicable hypotheses from the 'hypotheses_template' that align with the scenario described in the comment. Format your response as: {"all_relevant_hypotheses": [{"hypothesis_key": "[Insert appropriate hypothesis key from the hypotheses_template here]", "explanation": "[Provide an explanation in your word here for why you selected this hypothesis key]"}, ...]}.

3. Determine the most relevant hypothesis from the 'hypotheses_template' based on the comment, and provide a response in the format: {"best_hypothesis_key": "[Insert the most appropriate hypothesis key from the hypotheses_template here]", "best_hypothesis_value": "[Provide an explanation in your word here for why you selected this hypothesis key]"}.

Combine the responses into a single JSON response structured as:

{

"task_1_response": "Explanation for stopping the treatment or medication",

"task_2_response": {"all_relevant_hypotheses": [{"hypothesis_key": "[Insert appropriate hypothesis key from the hypotheses_template here]", "explanation": "[Provide an explanation in your word here for why you selected this hypothesis key]"}, ...]},

"task_3_response": {"best_hypothesis_key": "[Insert the most appropriate hypothesis key from the hypotheses_template here]", "best_hypothesis_value": "[Provide an explanation in your word here for why you selected this hypothesis key]"}

}

Enclosed 'hypotheses_template' JSON:

{

"Treatment Success": "The comment indicates that the patient discontinued the medication or treatment because their health condition improved significantly or the treatment course was successfully completed, rendering further treatment unnecessary.",

"Treatment Inefficacy": "The comment states that the medication or treatment was discontinued because it was not effective, compelling cessation of use.",

"Adverse Reactions": "This comment describes the discontinuation of medication or treatment due to adverse side effects, allergic reactions, or harmful interactions.",

"Accessibility Issues": "The comment indicates that the medication or treatment was stopped due to accessibility issues such as lack of insurance, financial constraints, market unavailability, or prescriber decisions.",

"Personal Choices": "The comment indicates that the medication or treatment was stopped based on personal choices, influenced by beliefs, lifestyle changes, or non-adherence.",

"Alternative Medical Reasons": "The comment indicates that the treatment was stopped for specific medical reasons not covered by other categories, such as pregnancy or a new health condition.",

"Indeterminate": "It is unclear why the medication or treatment was stopped.",

"Non-Discontinuation": "The comment does not indicate that any medication or treatment was stopped."

}

**Begin Analysis**

[TEXT GOES HERE]

**End Analysis**

“””

## Additional Results from DDE Detection

The tables below contain additional results regarding the performance of classifiers at the task of DDE detection. These tables provide detailed insights into various performance metrics across different classifiers and classification strategies.

**Table S1: ROC AUC and PR AUC for Classifiers and Classification Strategies**

Table S1 presents the Receiver Operating Characteristic (ROC) Area Under the Curve (AUC) and Precision-Recall (PR) AUC values for various classifiers under different classification strategies (CS1 and CS2). These metrics help evaluate the overall performance and discriminative power of the classifiers in identifying DDEs from the MedHelp.org dataset. The highest ROC AUC and PR AUC values were achieved by BART under CS1, demonstrating its superior performance in this context.

Table S1: ROC AUC and PR AUC for Classifiers and Classification Strategies

| Classifier | Strategy | ROC AUC | PR AUC |
| --- | --- | --- | --- |
| DistilRoBERTa | CS1 | 0.9008 | 0.6141 |
| DistilRoBERTa | CS2 | 0.8243 | 0.4430 |
| DistilBERT | CS1 | 0.8121 | 0.3308 |
| DistilBERT | CS2 | 0.6521 | 0.2626 |
| DeBERTa | CS1 | 0.9764 | 0.8239 |
| DeBERTa | CS2 | 0.9300 | 0.7204 |
| RoBERTa | CS1 | 0.9755 | 0.8114 |
| RoBERTa | CS2 | 0.8619 | 0.6752 |
| BART | CS1 | **0.9774** | **0.9043** |
| BART | CS2 | 0.9176 | 0.7087 |
| GPT3.5 | CS1 | 0.7337 | 0.2375 |
| GPT3.5 | CS2 | 0.6106 | 0.2151 |
| GPT4 | CS1 | 0.8892 | 0.6509 |
| GPT4 | CS2 | 0.8817 | 0.7078 |

1 The values represent the Receiver Operating Characteristics (ROC) Area Under Curve (AUC) and Precision-Recall (PR) AUC performance of different classifiers under different strategies. The highest ROC AUC and PR AUC were achieved by BART under CS1 and are shown in bold.

**Table S2: F1 Scores for Classifiers under CS1**

Table S2 details the F1 scores of different classifiers under the CS1 strategy at various cutoff values. The F1 score balances precision and recall, providing a comprehensive measure of a classifier's performance. BART achieved the highest F1 score of 0.86207 with a cutoff of 0.9, indicating its effectiveness in accurately identifying DDEs with minimal false positives and false negatives.

Table S2: F_1_ Scores for Classifiers under CS1

| Cutoff | RoBERTa | DistilRoBERTa | DeBERTa | DistilBERT | BART | GPT-3.5-  Turbo | GPT-4 |
| --- | --- | --- | --- | --- | --- | --- | --- |
| 0.05 | 0.20000 | 0.24971 | 0.23831 | 0.20452 | 0.21594 | 0.29720 | 0.36742 |
| 0.10 | 0.20557 | 0.30365 | 0.26355 | 0.20676 | 0.22961 | 0.33778 | 0.38416 |
| 0.15 | 0.21105 | 0.35069 | 0.29076 | 0.21010 | 0.25117 | 0.33929 | 0.39351 |
| 0.20 | 0.22408 | 0.39683 | 0.31845 | 0.21290 | 0.27296 | 0.34660 | 0.40842 |
| 0.25 | 0.24099 | 0.41758 | 0.34022 | 0.21599 | 0.29640 | 0.34906 | 0.41453 |
| 0.30 | 0.26161 | 0.45036 | 0.36770 | 0.21872 | 0.32132 | 0.34951 | 0.43016 |
| 0.35 | 0.28047 | 0.48677 | 0.39266 | 0.22438 | 0.35082 | 0.35036 | 0.42857 |
| 0.40 | 0.30141 | 0.49573 | 0.42126 | 0.22832 | 0.37857 | 0.35644 | 0.43636 |
| 0.45 | 0.32572 | 0.49850 | 0.44215 | 0.23450 | 0.42887 | 0.35644 | 0.43836 |
| 0.50 | 0.34684 | 0.51757 | 0.47345 | 0.23908 | 0.48148 | 0.37714 | 0.46715 |
| 0.55 | 0.37809 | 0.51178 | 0.49309 | 0.24150 | 0.52020 | 0.37714 | 0.46715 |
| 0.60 | 0.40684 | 0.51748 | 0.51566 | 0.24697 | 0.55495 | 0.38671 | 0.47525 |
| 0.65 | 0.43892 | 0.50185 | 0.53299 | 0.25313 | 0.61398 | **0.39144** | 0.48000 |
| 0.70 | 0.46799 | 0.54310 | 0.55703 | 0.26042 | 0.65359 | 0.36620 | 0.51337 |
| 0.75 | 0.50971 | **0.55721** | 0.59155 | 0.26991 | 0.71174 | 0.37692 | 0.51507 |
| 0.80 | 0.56911 | 0.55385 | 0.63444 | 0.28249 | 0.76336 | 0.38596 | 0.54819 |
| 0.85 | 0.60896 | 0.51685 | 0.67524 | 0.28994 | 0.80321 | 0.39091 | 0.55063 |
| 0.90 | 0.66887 | 0.50299 | **0.74733** | 0.30577 | **0.86207** | 0.35632 | 0.57937 |
| 0.95 | **0.73485** | 0.45033 | 0.73600 | **0.33696** | 0.85854 | 0.23077 | **0.61765** |

1 Of all the classifiers, BART under CS1 achieved the highest F_1_ score of 0.86207 with a cutoff of 0.9. The highest F_1_ score for each classifier is indicated in bold font

**Table S3: F1 Scores for Classifiers under CS2**

Table S3 shows the F1 scores of classifiers under the CS2 strategy, again at different cutoff values. The table highlights that DeBERTa performed best under CS2 with an F1 score of 0.68817 at a cutoff of 0.8, illustrating the classifier's capability to handle the combined context of multiple sentences.

Table S3: F_1_ Scores for Classifiers under CS2

| Cutoff | RoBERTa | DistilRoBERTa | DeBERTa | DistilBERT | BART | GPT-3.5-  Turbo | GPT-4 |
| --- | --- | --- | --- | --- | --- | --- | --- |
| 0.05 | 0.24350 | 0.37642 | 0.41860 | 0.22293 | 0.41336 | **0.22222** | 0.47644 |
| 0.10 | 0.27684 | 0.39355 | 0.48042 | 0.22957 | 0.50000 | 0.20571 | 0.49727 |
| 0.15 | 0.31333 | **0.41107** | 0.53529 | 0.22993 | 0.56522 | 0.21176 | 0.50276 |
| 0.20 | 0.33333 | 0.41071 | 0.56592 | 0.22889 | 0.60627 | 0.20513 | 0.51412 |
| 0.25 | 0.38261 | 0.40201 | 0.57732 | 0.23137 | 0.62992 | 0.20513 | 0.51412 |
| 0.30 | 0.41397 | 0.39153 | 0.59124 | 0.22495 | 0.65823 | 0.20779 | 0.51852 |
| 0.35 | 0.44759 | 0.38462 | 0.60536 | 0.23028 | **0.66667** | 0.20779 | 0.52299 |
| 0.40 | 0.49524 | 0.37931 | 0.63158 | 0.23789 | 0.65753 | 0.20779 | 0.53061 |
| 0.45 | 0.54355 | 0.39521 | 0.63025 | 0.24146 | 0.65116 | 0.20779 | 0.52632 |
| 0.50 | 0.57034 | 0.40000 | 0.65487 | 0.24703 | 0.65072 | 0.13636 | 0.53453 |
| 0.55 | 0.57600 | 0.37662 | 0.66977 | 0.25123 | 0.65672 | 0.13636 | 0.53614 |
| 0.60 | 0.61207 | 0.35762 | 0.66990 | 0.25000 | 0.65657 | 0.13636 | 0.54601 |
| 0.65 | 0.62385 | 0.36486 | 0.66337 | 0.24806 | 0.65979 | 0.13636 | 0.54769 |
| 0.70 | 0.62439 | 0.35862 | 0.67708 | 0.25337 | **0.66667** | 0.10938 | 0.56151 |
| 0.75 | **0.64322** | 0.31206 | 0.68449 | 0.25140 | 0.65574 | 0.11024 | 0.57605 |
| 0.80 | 0.63102 | 0.29630 | **0.68817** | 0.25595 | 0.64045 | 0.08333 | 0.60068 |
| 0.85 | 0.62857 | 0.23810 | 0.63218 | 0.26168 | 0.58683 | 0.08403 | 0.61314 |
| 0.90 | 0.58537 | 0.19672 | 0.59756 | 0.28188 | 0.52903 | 0.03571 | 0.64435 |
| 0.95 | 0.53333 | 0.08850 | 0.54902 | **0.29008** | 0.40845 | 0.03670 | **0.68342** |

1 Of all the classifiers, DeBERTa under CS2 achieved the highest F_1_ score of 0.68817 with a cutoff of 0.8. The highest F_1_ score for each classifier is indicated in bold font.

**Table S4: The Accuracy of Classifiers under CS1**

Table S4 provides the accuracy rates of various classifiers under CS1 across a range of cutoff values. Accuracy measures the proportion of true results (both true positives and true negatives) among the total number of cases examined. BART achieved the highest accuracy of 96.8% with a cutoff of 0.9, demonstrating its precision in detecting DDEs.

Table S4: The Accuracy of Classifiers under CS1

| Cutoff | RoBERTa | DistilRoBERTa | DeBERTa | DistilBERT | BART | GPT-3.5-  Turbo | GPT-4 |
| --- | --- | --- | --- | --- | --- | --- | --- |
| 0.05 | 0.144 | 0.357 | 0.316 | 0.191 | 0.223 | 0.598 | 0.666 |
| 0.10 | 0.173 | 0.523 | 0.402 | 0.202 | 0.282 | 0.702 | 0.689 |
| 0.15 | 0.200 | 0.626 | 0.478 | 0.218 | 0.362 | 0.704 | 0.701 |
| 0.20 | 0.259 | 0.696 | 0.542 | 0.231 | 0.430 | 0.721 | 0.719 |
| 0.25 | 0.326 | 0.735 | 0.585 | 0.245 | 0.492 | 0.724 | 0.726 |
| 0.30 | 0.396 | 0.773 | 0.632 | 0.257 | 0.548 | 0.732 | 0.743 |
| 0.35 | 0.451 | 0.806 | 0.669 | 0.281 | 0.604 | 0.733 | 0.744 |
| 0.40 | 0.504 | 0.823 | 0.706 | 0.297 | 0.652 | 0.740 | 0.752 |
| 0.45 | 0.557 | 0.833 | 0.730 | 0.321 | 0.723 | 0.740 | 0.754 |
| 0.50 | 0.597 | 0.849 | 0.762 | 0.338 | 0.776 | 0.782 | 0.781 |
| 0.55 | 0.648 | 0.855 | 0.780 | 0.353 | 0.810 | 0.782 | 0.781 |
| 0.60 | 0.688 | 0.862 | 0.799 | 0.378 | 0.838 | 0.797 | 0.788 |
| 0.65 | 0.729 | 0.865 | 0.816 | 0.404 | 0.873 | **0.801** | 0.792 |
| 0.70 | 0.759 | 0.894 | 0.833 | 0.432 | 0.894 | 0.820 | 0.818 |
| 0.75 | 0.798 | **0.911** | 0.855 | 0.459 | 0.919 | 0.838 | 0.823 |
| 0.80 | 0.841 | 0.913 | 0.879 | 0.492 | 0.938 | 0.860 | 0.850 |
| 0.85 | 0.869 | 0.914 | 0.899 | 0.520 | 0.951 | 0.866 | 0.858 |
| 0.90 | 0.900 | 0.917 | **0.929** | 0.555 | **0.968** | 0.888 | 0.894 |
| 0.95 | **0.930** | 0.917 | 0.934 | **0.634** | 0.971 | 0.900 | **0.922** |

1 Of all classifiers, BART achieved the highest accuracy of 96.8% under CS1 with a cutoff of

0.9. The cutoff corresponding to the highest F_1_ score achieved by each classifier is indicated in bold font.

**Table S5: The Accuracy of Classifiers under CS2**

Table S5 lists the accuracy rates for classifiers under CS2. DeBERTa reached the highest accuracy of 94.2% with a cutoff of 0.8, indicating its strong performance in a setting where multiple sentences are considered together for DDE detection.

Table S5: The Accuracy of Classifiers under CS2

| Cutoff | RoBERTa | DistilRoBERTa | DeBERTa | DistilBERT | BART | GPT-3.5-  Turbo | GPT-4 |
| --- | --- | --- | --- | --- | --- | --- | --- |
| 0.05 | 0.360 | 0.725 | 0.725 | 0.512 | 0.719 | **0.846** | 0.800 |
| 0.10 | 0.488 | 0.812 | 0.801 | 0.557 | 0.814 | 0.861 | 0.816 |
| 0.15 | 0.588 | **0.851** | 0.842 | 0.578 | 0.860 | 0.866 | 0.820 |
| 0.20 | 0.644 | 0.868 | 0.865 | 0.589 | 0.887 | 0.876 | 0.828 |
| 0.25 | 0.716 | 0.881 | 0.877 | 0.608 | 0.906 | 0.876 | 0.828 |
| 0.30 | 0.765 | 0.885 | 0.888 | 0.621 | 0.919 | 0.878 | 0.831 |
| 0.35 | 0.805 | 0.888 | 0.897 | 0.639 | **0.924** | 0.878 | 0.834 |
| 0.40 | 0.841 | 0.892 | 0.909 | 0.654 | 0.925 | 0.878 | 0.839 |
| 0.45 | 0.869 | 0.899 | 0.912 | 0.667 | 0.925 | 0.878 | 0.838 |
| 0.50 | 0.887 | 0.904 | 0.922 | 0.683 | 0.927 | 0.886 | 0.845 |
| 0.55 | 0.894 | 0.904 | 0.929 | 0.696 | 0.931 | 0.886 | 0.846 |
| 0.60 | 0.910 | 0.903 | 0.932 | 0.700 | 0.932 | 0.886 | 0.852 |
| 0.65 | 0.918 | 0.906 | 0.932 | 0.709 | 0.934 | 0.886 | 0.853 |
| 0.70 | 0.923 | 0.907 | 0.938 | 0.723 | **0.937** | 0.886 | 0.861 |
| 0.75 | **0.929** | 0.903 | 0.941 | 0.732 | 0.937 | 0.887 | 0.869 |
| 0.80 | 0.931 | 0.905 | **0.942** | 0.750 | 0.936 | 0.890 | 0.883 |
| 0.85 | 0.935 | 0.904 | 0.936 | 0.763 | 0.931 | 0.891 | 0.894 |
| 0.90 | 0.932 | 0.902 | 0.934 | 0.786 | 0.927 | 0.892 | 0.915 |
| 0.95 | 0.930 | 0.897 | 0.931 | **0.814** | 0.916 | 0.895 | **0.937** |

1 Of all classifiers, DeBERTa achieved the highest accuracy of 94.2% under CS2 with a cutoff of 0.8. The cutoff corresponding to the highest F_1_ score achieved by each classifier is indicated in bold font.

**Table S6: False Positive Rates (FPRs) of Classifiers under CS1**

Table S6 presents the False Positive Rates (FPRs) for different classifiers under CS1. The FPR measures the proportion of negative instances that were incorrectly classified as positive. BART maintained a low FPR, showcasing its ability to minimize false alarms while accurately detecting DDEs.

Table S6: False Positive Rates (FPRs) of Classifiers under CS1

| Cutoff | RoBERTa | DistilRoBERTa | DeBERTa | DistilBERT | BART | GPT-3.5-  Turbo | GPT-4 |
| --- | --- | --- | --- | --- | --- | --- | --- |
| 0.05 | 0.95857 | 0.72004 | 0.76596 | 0.90258 | 0.87010 | 0.42553 | 0.36282 |
| 0.10 | 0.92609 | 0.53080 | 0.66965 | 0.89026 | 0.80403 | 0.29899 | 0.33707 |
| 0.15 | 0.89586 | 0.41209 | 0.58455 | 0.87234 | 0.71445 | 0.29675 | 0.32363 |
| 0.20 | 0.82979 | 0.33259 | 0.51288 | 0.85778 | 0.63830 | 0.27548 | 0.30347 |
| 0.25 | 0.75476 | 0.28331 | 0.46473 | 0.84211 | 0.56887 | 0.27212 | 0.29563 |
| 0.30 | 0.67637 | 0.23852 | 0.41209 | 0.82867 | 0.50616 | 0.26092 | 0.27660 |
| 0.35 | 0.61478 | 0.20045 | 0.37066 | 0.80179 | 0.44345 | 0.25980 | 0.27436 |
| 0.40 | 0.55543 | 0.17581 | 0.32923 | 0.78387 | 0.38858 | 0.25196 | 0.26540 |
| 0.45 | 0.49608 | 0.16013 | 0.30235 | 0.75700 | 0.30683 | 0.25196 | 0.26316 |
| 0.50 | 0.45129 | 0.13998 | 0.26652 | 0.73796 | 0.24748 | 0.19821 | 0.23292 |
| 0.55 | 0.39418 | 0.12766 | 0.24636 | 0.72004 | 0.20829 | 0.19821 | 0.23292 |
| 0.60 | 0.34938 | 0.11758 | 0.22508 | 0.69093 | 0.17469 | 0.17917 | 0.22508 |
| 0.65 | 0.30235 | 0.10750 | 0.20381 | 0.66069 | 0.13550 | **0.17469** | 0.22060 |
| 0.70 | 0.26876 | 0.06943 | 0.18477 | 0.62822 | 0.11086 | 0.13998 | 0.19149 |
| 0.75 | 0.22396 | **0.04255** | 0.16013 | 0.59798 | 0.08287 | 0.11646 | 0.18365 |
| 0.80 | 0.17581 | 0.03807 | 0.13326 | 0.56103 | 0.06159 | 0.08623 | 0.15006 |
| 0.85 | 0.14110 | 0.02800 | 0.11086 | 0.52744 | 0.04703 | 0.07839 | 0.13662 |
| 0.90 | 0.10526 | 0.02016 | **0.07727** | 0.48824 | **0.02800** | 0.04031 | 0.08063 |
| 0.95 | **0.06719** | 0.01120 | 0.05711 | **0.39418** | 0.01120 | 0.00896 | **0.03807** |

**Table S7: False Positive Rates (FPRs) of Classifiers under CS2**

Table S7 shows the FPRs for classifiers under CS2, where BART again demonstrated lower false positive rates compared to other classifiers, underlining its reliability in different contexts.

Table S7: False Positive Rates (FPRs) of Classifiers under CS2

| Cutoff | RoBERTa | DistilRoBERTa | DeBERTa | DistilBERT | BART | GPT-3.5-  Turbo | GPT-4 |
| --- | --- | --- | --- | --- | --- | --- | --- |
| 0.05 | 0.71221 | 0.28108 | 0.29899 | 0.50504 | 0.30571 | **0.07727** | 0.20605 |
| 0.10 | 0.56327 | 0.15901 | 0.20605 | 0.45017 | 0.19261 | 0.05599 | 0.18813 |
| 0.15 | 0.44681 | **0.10526** | 0.15901 | 0.42329 | 0.13886 | 0.05039 | 0.18365 |
| 0.20 | 0.37850 | 0.07951 | 0.12990 | 0.40873 | 0.10414 | 0.03695 | 0.17469 |
| 0.25 | 0.29675 | 0.05823 | 0.11198 | 0.38522 | 0.07503 | 0.03695 | 0.17469 |
| 0.30 | 0.23628 | 0.05039 | 0.09630 | 0.36618 | 0.05823 | 0.03471 | 0.17133 |
| 0.35 | 0.18701 | 0.04479 | 0.08399 | 0.34490 | **0.05039** | 0.03471 | 0.16797 |
| 0.40 | 0.14558 | 0.03807 | 0.06943 | 0.32811 | 0.04479 | 0.03471 | 0.16237 |
| 0.45 | 0.11422 | 0.03024 | 0.06271 | 0.31243 | 0.04255 | 0.03471 | 0.16237 |
| 0.50 | 0.09071 | 0.02352 | 0.05039 | 0.29339 | 0.03807 | 0.01792 | 0.15342 |
| 0.55 | 0.07951 | 0.02016 | 0.04031 | 0.27772 | 0.03135 | 0.01792 | 0.15230 |
| 0.60 | 0.06047 | 0.01904 | 0.03359 | 0.27212 | 0.02912 | 0.01792 | 0.14558 |
| 0.65 | 0.04815 | 0.01568 | 0.03135 | 0.25980 | 0.02576 | 0.01792 | 0.14446 |
| 0.70 | 0.03807 | 0.01344 | 0.02240 | 0.24300 | **0.02128** | 0.01568 | 0.13550 |
| 0.75 | **0.03135** | 0.01344 | 0.01792 | 0.23068 | 0.01792 | 0.01456 | 0.12654 |
| 0.80 | 0.02352 | 0.00896 | **0.01680** | 0.20829 | 0.01568 | 0.00896 | 0.10974 |
| 0.85 | 0.01456 | 0.00448 | 0.01344 | 0.19261 | 0.01232 | 0.00784 | 0.09295 |
| 0.90 | 0.01008 | 0.00336 | 0.00896 | 0.16685 | 0.00784 | 0.00336 | 0.06159 |
| 0.95 | 0.00336 | 0.00112 | 0.00448 | **0.13102** | 0.00672 | 0.00000 | **0.02688** |

**Table S8: False Negative Rates (FNRs) for Classifiers under CS1**

Table S8 details the False Negative Rates (FNRs) of classifiers under CS1. The FNR indicates the proportion of positive instances that were incorrectly classified as negative. The table highlights how each classifier performed in terms of missing actual DDEs.

Table S8: False Negative Rate (FNR) for Classifiers under CS1

| Cutoff | RoBERTa | DistilRoBERTa | DeBERTa | DistilBERT | BART | GPT-3.5-  Turbo | GPT-4 |
| --- | --- | --- | --- | --- | --- | --- | --- |
| 0.05 | 0.00000 | 0.00000 | 0.00000 | 0.02804 | 0.00000 | 0.20561 | 0.09346 |
| 0.10 | 0.00000 | 0.02804 | 0.00000 | 0.02804 | 0.00000 | 0.28972 | 0.09346 |
| 0.15 | 0.00000 | 0.05607 | 0.00000 | 0.02804 | 0.00000 | 0.28972 | 0.09346 |
| 0.20 | 0.00000 | 0.06542 | 0.00000 | 0.02804 | 0.00000 | 0.30841 | 0.09346 |
| 0.25 | 0.00000 | 0.11215 | 0.00000 | 0.02804 | 0.00000 | 0.30841 | 0.09346 |
| 0.30 | 0.00000 | 0.13084 | 0.00000 | 0.02804 | 0.00000 | 0.32710 | 0.09346 |
| 0.35 | 0.00000 | 0.14019 | 0.00000 | 0.02804 | 0.00000 | 0.32710 | 0.10280 |
| 0.40 | 0.00000 | 0.18692 | 0.00000 | 0.02804 | 0.00935 | 0.32710 | 0.10280 |
| 0.45 | 0.00000 | 0.22430 | 0.00000 | 0.02804 | 0.02804 | 0.32710 | 0.10280 |
| 0.50 | 0.00000 | 0.24299 | 0.00000 | 0.02804 | 0.02804 | 0.38318 | 0.10280 |
| 0.55 | 0.00000 | 0.28972 | 0.00000 | 0.03738 | 0.03738 | 0.38318 | 0.10280 |
| 0.60 | 0.00000 | 0.30841 | 0.00000 | 0.04673 | 0.05607 | 0.40187 | 0.10280 |
| 0.65 | 0.00935 | 0.36449 | 0.01869 | 0.05607 | 0.05607 | **0.40187** | 0.10280 |
| 0.70 | 0.00935 | 0.41121 | 0.01869 | 0.06542 | 0.06542 | 0.51402 | 0.10280 |
| 0.75 | 0.01869 | **0.47664** | 0.01869 | 0.06542 | 0.06542 | 0.54206 | 0.12150 |
| 0.80 | 0.01869 | 0.49533 | 0.01869 | 0.06542 | 0.06542 | 0.58879 | 0.14953 |
| 0.85 | 0.04673 | 0.57009 | 0.01869 | 0.08411 | 0.06542 | 0.59813 | 0.18692 |
| 0.90 | 0.05607 | 0.60748 | **0.01869** | 0.08411 | **0.06542** | 0.71028 | 0.31776 |
| 0.95 | **0.09346** | 0.68224 | 0.14019 | **0.13084** | 0.17757 | 0.85981 | **0.41121** |

**Table S9: False Negative Rates (FNRs) for Classifiers under CS2**

Table S9 provides the FNRs for classifiers under CS2. This table is essential for understanding the instances where classifiers failed to detect actual DDEs, emphasizing the importance of balancing sensitivity and specificity.

Table S9: False Negative Rate (FNR) for Classifiers under CS2

| Cutoff | RoBERTa | DistilRoBERTa | DeBERTa | DistilBERT | BART | GPT-3.5-  Turbo | GPT-4 |
| --- | --- | --- | --- | --- | --- | --- | --- |
| 0.05 | 0.03738 | 0.22430 | 0.07477 | 0.34579 | 0.07477 | **0.79439** | 0.14953 |
| 0.10 | 0.08411 | 0.42991 | 0.14019 | 0.38318 | 0.13084 | 0.83178 | 0.14953 |
| 0.15 | 0.12150 | **0.51402** | 0.14953 | 0.41121 | 0.14953 | 0.83178 | 0.14953 |
| 0.20 | 0.16822 | 0.57009 | 0.17757 | 0.42991 | 0.18692 | 0.85047 | 0.14953 |
| 0.25 | 0.17757 | 0.62617 | 0.21495 | 0.44860 | 0.25234 | 0.85047 | 0.14953 |
| 0.30 | 0.22430 | 0.65421 | 0.24299 | 0.48598 | 0.27103 | 0.85047 | 0.14953 |
| 0.35 | 0.26168 | 0.67290 | 0.26168 | 0.49533 | **0.28972** | 0.85047 | 0.14953 |
| 0.40 | 0.27103 | 0.69159 | 0.27103 | 0.49533 | 0.32710 | 0.85047 | 0.14953 |
| 0.45 | 0.27103 | 0.69159 | 0.29907 | 0.50467 | 0.34579 | 0.85047 | 0.15888 |
| 0.50 | 0.29907 | 0.70093 | 0.30841 | 0.51402 | 0.36449 | 0.91589 | 0.16822 |
| 0.55 | 0.32710 | 0.72897 | 0.32710 | 0.52336 | 0.38318 | 0.91589 | 0.16822 |
| 0.60 | 0.33645 | 0.74766 | 0.35514 | 0.53271 | 0.39252 | 0.91589 | 0.16822 |
| 0.65 | 0.36449 | 0.74766 | 0.37383 | 0.55140 | 0.40187 | 0.91589 | 0.16822 |
| 0.70 | 0.40187 | 0.75701 | 0.39252 | 0.56075 | **0.41121** | 0.93458 | 0.16822 |
| 0.75 | **0.40187** | 0.79439 | 0.40187 | 0.57944 | 0.43925 | 0.93458 | 0.16822 |
| 0.80 | 0.44860 | 0.81308 | **0.40187** | 0.59813 | 0.46729 | 0.95327 | 0.17757 |
| 0.85 | 0.48598 | 0.85981 | 0.48598 | 0.60748 | 0.54206 | 0.95327 | 0.21495 |
| 0.90 | 0.55140 | 0.88785 | 0.54206 | 0.60748 | 0.61682 | 0.98131 | 0.28037 |
| 0.95 | 0.62617 | 0.95327 | 0.60748 | **0.64486** | 0.72897 | 0.98131 | **0.36449** |
